# Supplementary material for: Epistasis lowers the genetic barrier to SARS-CoV-2 neutralizing antibody escape
Source: bioRxiv. 2022 Aug 19:2022.08.17.504313. Preprint. [Version 1] doi: 10.1101/2022.08.17.504313 (PMC9413706; doi:10.1101/2022.08.17.504313)
Supplement: 1 [file NIHPP2022.08.17.504313v1-supplement-1.pdf]

bioRxiv preprint doi: https://doi.org/10.1101/2022.08.17.504313; this version posted August 19, 2022. The copyright holder for this preprint (which was not certified by peer review) is the author/funder, who has granted bioRxiv a license to display the preprint in perpetuity. It is made available under aCC-BY 4.0 International license.

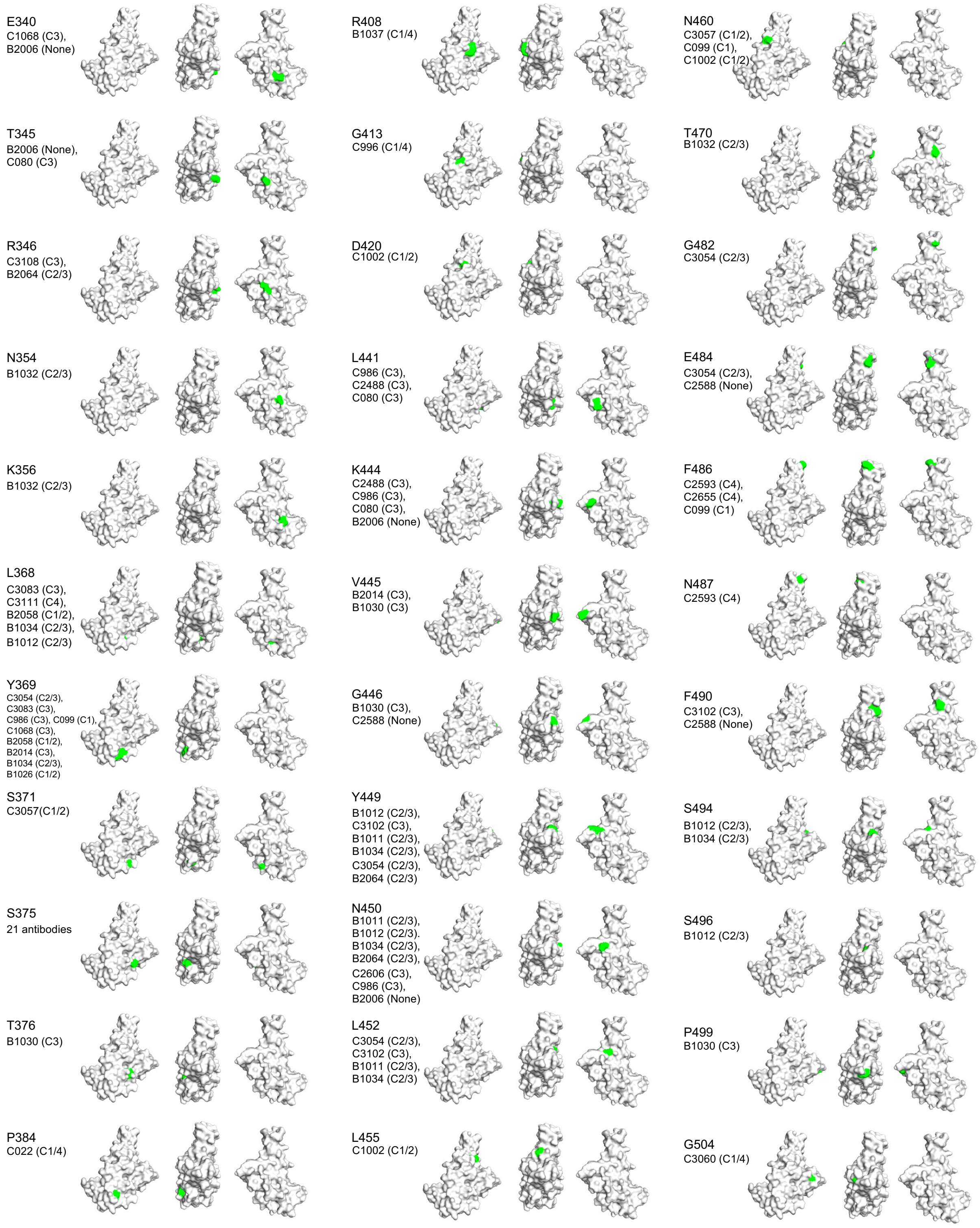

Supplementary Figure 1 Substitutions enriched in rVSV/SARS-CoV-2 populations selected by broadly neutralizing antibodies

a, RBD structure (PDB ID : 7C8J) with positions (highlighted in green), at which substitutions occurring at frequencies of >10% were found after two passages of rVSV/SARS-CoV-2 encoding Wuhan-Hu-1, BA.1, and BA.2 spike proteins in the presence of 1 µg/ml of the indicated broadly neutralizing antibody whose class 1-4 designation is indicated.

bioRxiv preprint doi: https://doi.org/10.1101/2022.08.17.504313; this version posted August 19, 2022. The copyright holder for this preprint (which was not certified by peer review) is the author/funder, who has granted bioRxiv a license to display the preprint in perpetuity. It is made available under aCC-BY 4.0 International license.

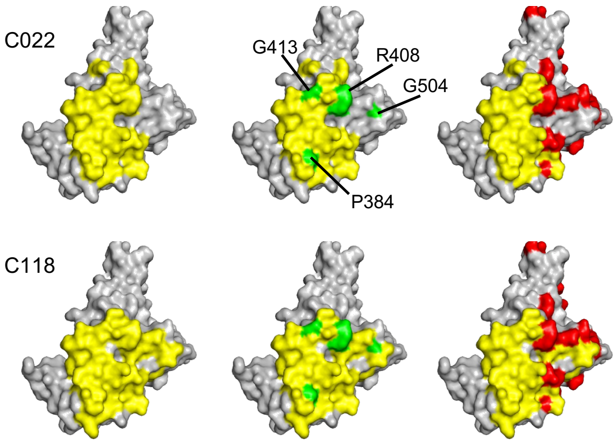

Supplementary Figure 2 Resistance to broadly neutralizing class 4 and 1/4 antibodies. RBD structure (PDB ID : 7C8J) illustrating epitopes of two prototype class 4 antibodies (C022 and C118, yellow), substitutions that confer resistance to class 4 and 1/4 antibodies (green), and preexisting substitutions in BA.2 (red)

bioRxiv preprint doi: https://doi.org/10.1101/2022.08.17.504313; this version posted August 19, 2022. The copyright holder for this preprint (which was not certified by peer review) is the author/funder, who has granted bioRxiv a license to display the preprint in perpetuity. It is made available under aCC-BY 4.0 International license.

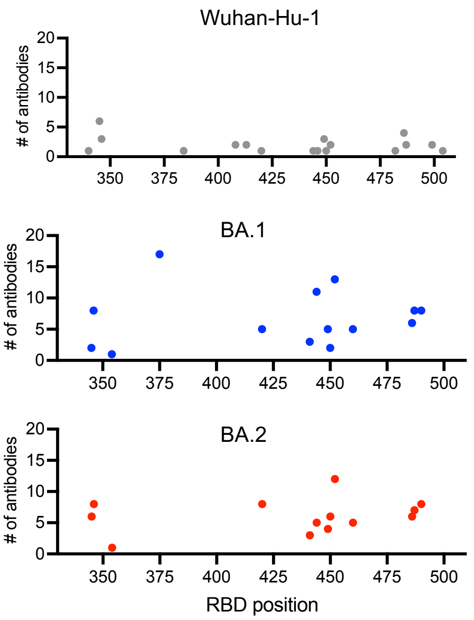

Supplementary Figure 3 Context dependent effects of RBD substitutions on antibody escape

Number of broadly neutralizing antibodies for which substitutions in Wuhan-Hu-1, BA.1, and BA.2 backgrounds at positions along the length of the RBD confer escape. Antibody escape was defined as >5-fold increase in mutant pseudotype relative infection compared to parental pseudotype in the presence of 1μg/ml antibody and >10% relative infection compared to no antibody.

bioRxiv preprint doi: https://doi.org/10.1101/2022.08.17.504313; this version posted August 19, 2022. The copyright holder for this preprint (which was not certified by peer review) is the author/funder, who has granted bioRxiv a license to display the preprint in perpetuity. It is made available under aCC-BY 4.0 International license.

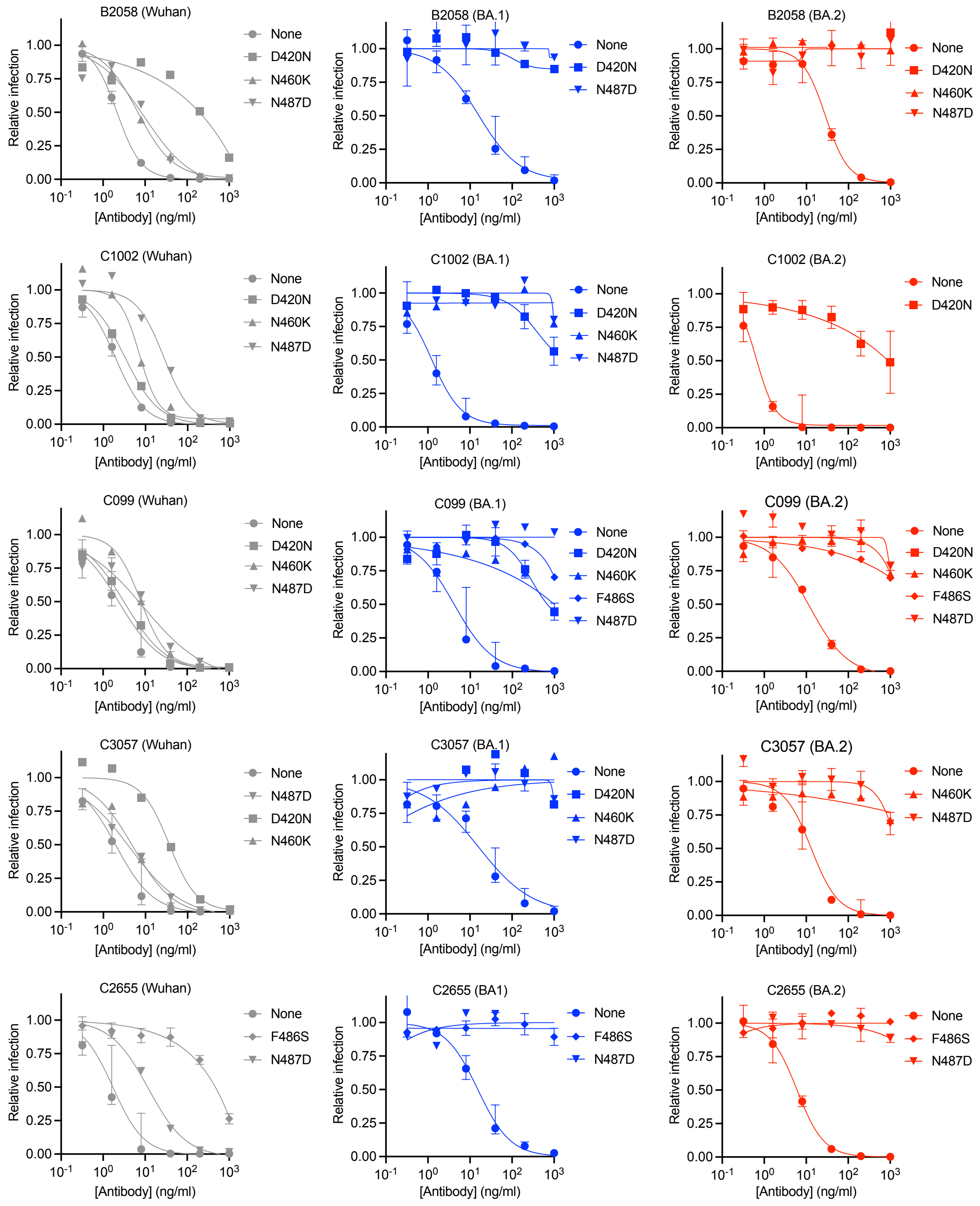

Supplementary Figure 4 Epistatic effects of substitutions on broadly neutralizing class 1, 1/2 and 1/4 antibodies

Neutralization of RBD point mutant pseudotypes in Wuhan-Hu-1, BA.1, and BA.2 backgrounds by class 1, 1/2 and 1/4 antibodies. Median and range of 2 or 3 independent experiments is shown

bioRxiv preprint doi: https://doi.org/10.1101/2022.08.17.504313; this version posted August 19, 2022. The copyright holder for this preprint (which was not certified by peer review) is the author/funder, who has granted bioRxiv a license to display the preprint in perpetuity. It is made available under aCC-BY 4.0 International license.

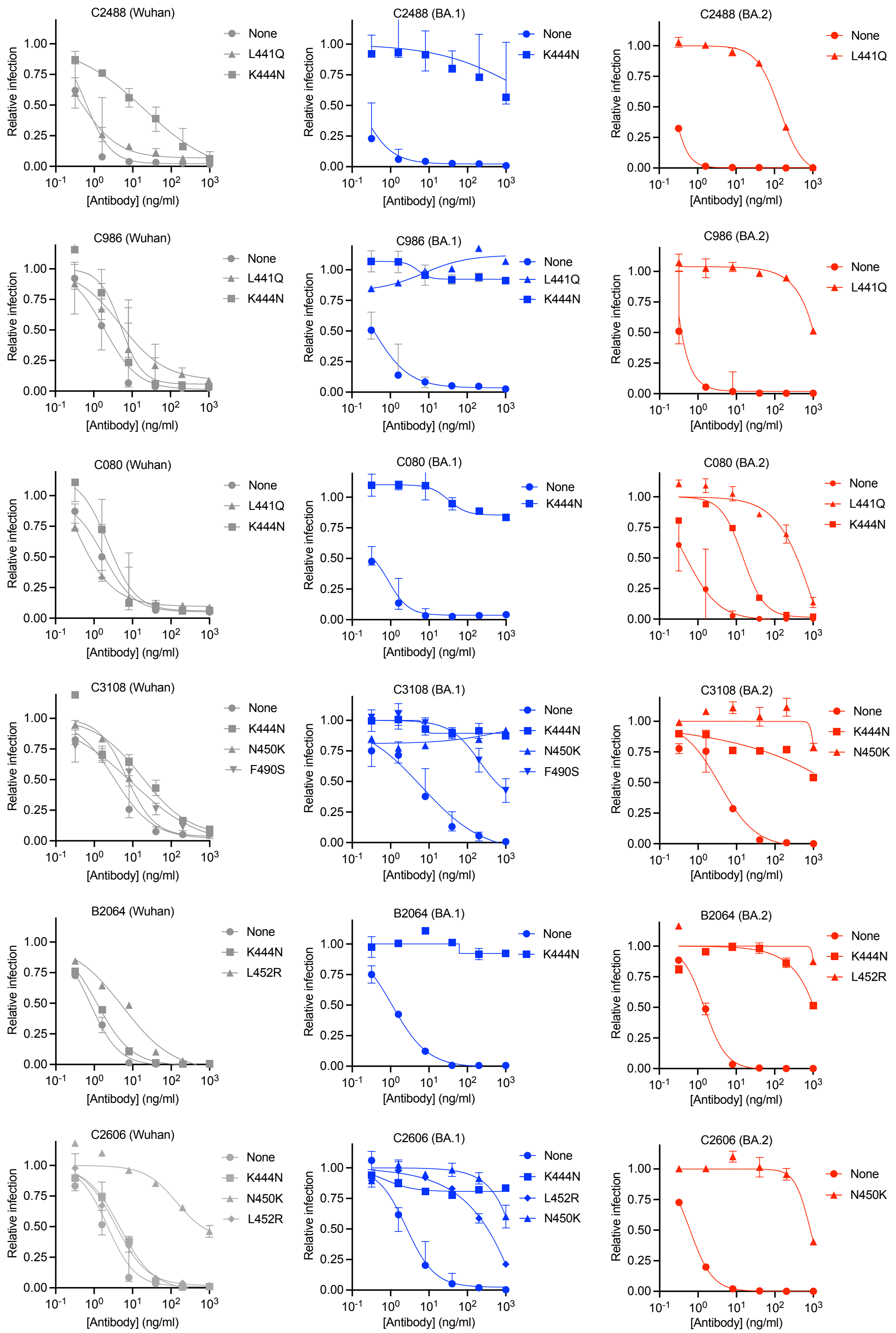

Supplementary 5 Epistatic effects of substitutions on broadly neutralizing class 2/3 and 3 antibodies (I)

Neutralization of RBD point mutant pseudotypes in Wuhan-Hu-1, BA.1, and BA.2 backgrounds by class 2/3 and 3 antibodies. Median and range of 2 or 3 independent experiments is shown

bioRxiv preprint doi: https://doi.org/10.1101/2022.08.17.504313; this version posted August 19, 2022. The copyright holder for this preprint (which was not certified by peer review) is the author/funder, who has granted bioRxiv a license to display the preprint in perpetuity. It is made available under aCC-BY 4.0 International license.

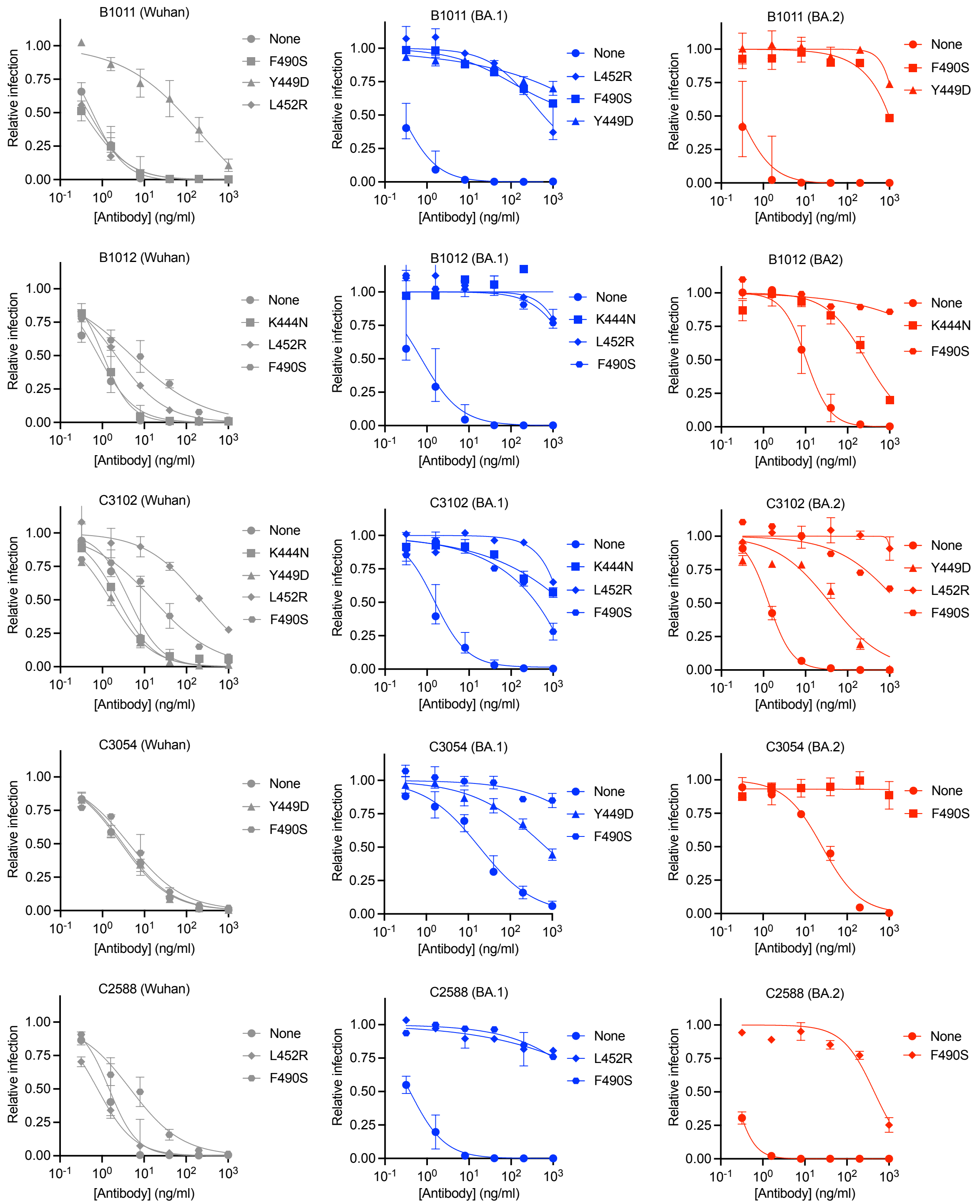

Supplementary 6 Epistatic effects of substitutions on broadly neutralizing class 2/3 and 3 antibodies (II)

Neutralization of RBD point mutant pseudotypes in Wuhan-Hu-1, BA.1, and BA.2 backgrounds by class 2/3 and 3 antibodies. Median and range of 2 or 3 independent experiments is shown

bioRxiv preprint doi: https://doi.org/10.1101/2022.08.17.504313; this version posted August 19, 2022. The copyright holder for this preprint (which was not certified by peer review) is the author/funder, who has granted bioRxiv a license to display the preprint in perpetuity. It is made available under aCC-BY 4.0 International license.

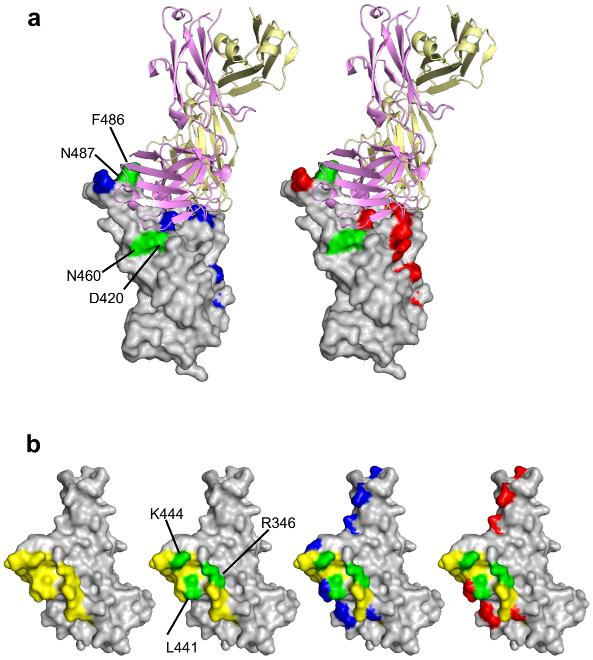

Supplementary Figure 7 Pre-existing and escape substitutions in broadly neutralizing class 1 and class 3 antibody epitopes

a) Substitutions that confer context-dependent escape from the class I antibody C099, depicted on the C099:RBD complex structure (PDB ID 7R8L). Green indicates C099 escape substitutions. Blue and red indicate BA.1 and BA.2 substitutions, respectively. C099 heavy and light chains are magenta and yellow respectively. **b)** Substitutions that confer context-dependent escape from C080 class 3 antibody, depicted on RBD complex structure (PDB ID 7C8J). Yellow indicates the C032 (clonal ancestor of C080) epitope. Green indicates escape substitutions. Blue and red indicate BA.1 and BA.2 substitutions, respectively.

bioRxiv preprint doi: https://doi.org/10.1101/2022.08.17.504313; this version posted August 19, 2022. The copyright holder for this preprint (which was not certified by peer review) is the author/funder, who has granted bioRxiv a license to display the preprint in perpetuity. It is made available under aCC-BY 4.0 International license.

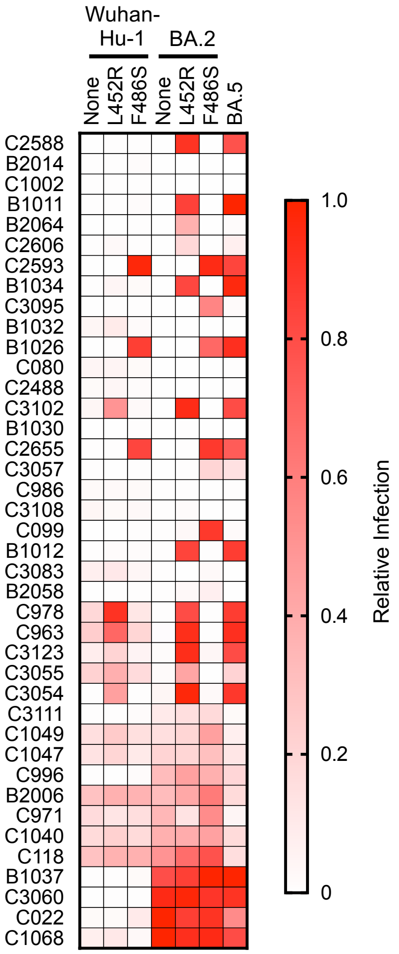

Supplementary Figure 8 Context dependent effect of BA.5 substitutions (L452 and F486) on broadly neutralizing antibody sensitivity

Inhibition of Wuhan-Hu-1 and BA.2 RBD pseudotypes with L452R or F486S substitutions by broadly neutralizing antibodies, in comparison with BA.5. Relative infection is defined as the decimal fraction of infection measured (with 1 µg/ml antibody), relative to an uninhibited virus control (without antibody). Antibodies are listed in descending order of potency against BA.2 and median values from two independent experiments are displayed.

bioRxiv preprint doi: https://doi.org/10.1101/2022.08.17.504313; this version posted August 19, 2022. The copyright holder for this preprint (which was not certified by peer review) is the author/funder, who has granted bioRxiv a license to display the preprint in perpetuity. It is made available under aCC-BY 4.0 International license.

Supplementary Data 1: Demographics and SARS-CoV-2 clinical histories of participants

Supplementary Data 2: Properties and sequences of broadly neutralizing monoclonal antibodies

Supplementary Data 3: Frequencies of RBD substitutions identified during antibody selection experiments

Supplementary Data 4: Neutralization of Wuhan-Hu-1, BA.1, and BA.2 RBD point mutant pseudotypes by broadly neutralizing antibodies

Supplementary Data 5: p-values for Figure 4
